# Cost-effectiveness analysis of metformin+dipeptidyl peptidase-4 inhibitors compared to metformin+sulfonylureas for treatment of type 2 diabetes

**DOI:** 10.1186/s12913-018-2860-0

**Published:** 2018-02-01

**Authors:** Christina S. Kwon, Enrique Seoane-Vazquez, Rosa Rodriguez-Monguio

**Affiliations:** 1 International Center for Pharmaceutical Economics & Policy, MCPHS University, 179 Longwood Ave, Boston, MA 02115-5804 USA; 2 Department of Biomedical and Pharmaceutical Sciences, Chapman University School of Pharmacy, Harry and Diane Rinker Health Science Campus RK 94-271, 9401 Jeronimo Road, Irvine, CA 92618-1908 USA; 3 Medication Outcomes Center, School of Pharmacy, University of California San Francisco, 533 Parnassus Avenue, San Francisco, CA 94143-0622 USA

**Keywords:** Cost-effectiveness analysis, Type 2 diabetes, Costs, Outcomes, Life years gained, Metformin, Sulfonylureas, Dipeptidyl peptidase-4 inhibitors

## Abstract

**Background:**

Patients with type 2 diabetes (T2D) typically use several drug treatments during their lifetime. There is a debate about the best second-line therapy after metformin monotherapy failure due to the increasing number of available antidiabetic drugs and the lack of comparative clinical trials of secondary treatment regimens. While prior research compared the cost-effectiveness of two alternative drugs, the literature assessing T2D treatment pathways is scarce. The purpose of this study was to evaluate the long-term cost-effectiveness of dipeptidyl peptidase-4 inhibitors (DPP-4i) compared to sulfonylureas (SU) as second-line therapy in combination with metformin in patients with T2D.

**Methods:**

A Markov model was developed with four health states, 1 year cycle, and a 25-year time horizon. Clinical and cost data were collected from previous studies and other readily available secondary data sources. The incremental cost-effectiveness ratio (ICER) was estimated from the US third party payer perspective. Both, costs and outcomes, were discounted at a 3% annual discount rate. One way and probabilistic sensitivity analyses were performed to evaluate the impact of uncertainty on the base-case results.

**Results:**

The discounted incremental cost of metformin+DPP-4i compared to metformin+SU was $11,849 and the incremental life-years gained were 0.61, resulting in an ICER of $19,420 per life-year gained for patients in the metformin+DPP-4i treatment pathway. The ICER estimated in the probabilistic sensitivity analysis was $19,980 per life-year gained. Sensitivity analyses showed that the results of the study were not sensitive to changes in the parameters used in base-case.

**Conclusions:**

The metformin+DPP-4i treatment pathway was cost-effective compared to metformin+SU as a long-term second-line therapy in the treatment of T2D from the US health care payer perspective. Study findings have the potential to provide clinicians and third party payers valuable evidence for the prescription and utilization of cost-effective second-line therapy after metformin monotherapy failure in the treatment of T2D.

## Background

Diabetes mellitus is one of the most prevalent and costly chronic diseases in the United States (US). In 2012, 9.3% of the US population had diabetes mellitus [1]. In that year 2012, the health care cost of diagnosed diabetes in the US totaled $245 billion [2]. The US market of antidiabetic products reached $43.9 billion in 2015 (a 109.0% increase from $21.0 billion in 2011) [3]. The number of prescriptions for antidiabetic drugs totaled 211 million in 2015 (compared to 174 million in 2011) [3]. In 2015, insulin glargine recombinant was the top fifth drug by sales in the US totaling $5.8 billion (241.2% increase compared to 2011) [3]. Sitagliptin was the top tenth prescription drug by sales reaching $4.2 billion in 2015 (a 90.9% increase compared to 2011) [3]. As of December 31, 2015, there were 27 unique non-insulin antidiabetic drugs, belonging to 12 therapeutic classes, including 5 modified formulations and 18 fixed-dose combinations of active ingredients, available in the US market [4].

Metformin has a well-established long-term post-marketing evidence of effectiveness and safety [5–7]. While there is a general consensus about the use of metformin as first-line therapy for type 2 diabetes (T2D) [5–7]; there is a vigorous debate about best second-line treatment regimen [8]. Sulfonylureas (SU) are a common second-line therapy due to their fast onset on blood glucose lowering [9, 10]. However, safety related concerns, including risk of hypoglycemia and weight gain, have been raised [9, 10]. Dipeptidyl peptidase-4 inhibitors (DPP-4i) are newer drugs with lower risk of hypoglycemia and weight gain but lower glycemic lowering effect than SU [10, 11]. In addition, DPP-4i are costlier than SU.

Two previous studies explored the cost-effectiveness of SU compared to DPP-4i as second-line therapy after metformin failure in the US. Study findings were inconclusive. Bergenheim et al. (2012) [12] assessed the lifetime cost-effectiveness of metformin+SU and metformin+DPP-4i in T2D using data from 52-week randomized controlled trial [9]. The authors concluded that DPP-4i was a cost-effective second-line therapy after metformin failure in the US. Zhang et al. (2014) [8] compared the medication cost and effectiveness of metformin+SU, DPP-4i, and glucagon-like peptide-1(GLP-1) receptor agonists as the second-line therapy until first diabetes-related complication or death. The authors found that metformin+SU resulted in similar outcomes but lower drug costs compared to other two comparators.

Bergenheim et al., (2012) did not consider insulin treatment after second-line failure; whereas, Zhang et al., (2014) included insulin treatment as third-line in their analyses. Furthermore, Bergenheim et al., (2012) included drug cost and diabetes related health care costs in their economic evaluation; whereas, Zhang et al., (2014) did not assess health care costs associated with diabetic complications, which often pose a significant economic burden on patients with T2D [1].

Additionally, a study conducted by Langer et al., (2013) [13] assessed the short-term cost-effectiveness of metformin+sitagliptin (i.e., DPP-4i inhibitor class) compared to metformin+liraglutide (i.e., GLP-1 receptor agonists) based on data derived from a 26-week randomized, controlled trial conducted by Pratley et al., (2010) [14]. The study time horizon was only 1 year. Authors found that mean cost per patient reaching target glycated hemoglobin (A1c) was lower for liraglutide than sitagliptin. Langer et al., (2013) included only drug costs in their analyses.

Prior research compared short-term cost-effectiveness of two alternative drugs for treatment of T2D. To the best of authors’ knowledge, no previous US studies assessed the cost-effectiveness of alternative T2D treatment pathways over a patient’s lifetime. Thus, this study assessed the long-term cost-effectiveness of dipeptidyl peptidase-4 inhibitors compared to sulfonylureas as second-line therapy for the treatment of T2D. This study has the potential to provide clinicians and third party payers with new perspectives on the cost-effectiveness of long-term treatment pathways for T2D.

## Methods

### Therapeutic alternatives

Most patients with T2D take one or more drugs in addition to metformin monotherapy to control their blood glucose levels and eventually, will initiate insulin therapy alone or in combination with other non-insulin antidiabetic drugs when previous alternatives fail. In the scenario analysis, there were two different treatments pathways. Both pathways started with metformin monotherapy, the most common treatment. Patient used metformin+DPP-4i or metformin+SU as second-line therapy when metformin monotherapy failed. In addition, both treatment pathways added basal insulin therapy in patients with T2D when combination therapy failed.

### Markov model

**Fig. 1:**
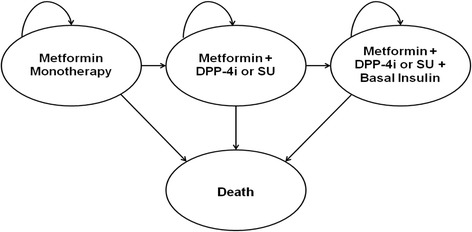
Markov Model Diagram, Acronyms: DPP-4i-dipeptidyl peptidase-4 inhibitors, SU-sulfonylureas

A Markov model constructed in Microsoft Excel 2013 software was based on current T2D treatment guidelines [5, 6]. The Markov model had four states (Fig. 1). In the first state, patients used metformin monotherapy. Patients could remain in the first state or transition to the second state. In the second state, either DPP-4i or SU was added to metformin as second-line therapy. Likewise, patients could remain in the second state or transition to the third state where basal insulin was added to their current therapy as third-line therapy. Patients in those three states could transition anytime to death (i.e., absorbing state). We assumed that patients initiated metformin monotherapy at age 60 years with a time horizon of 25 years (i.e., 60–85 years old). The cycle duration was 1 year [15].

### Health outcomes and cost

Health outcomes data were collected from the literature (Table 1). Treatment failure rates obtained from previous studies were used to determine the annual probability of transitioning from metformin monotherapy to oral antidiabetic drug (OAD) dual therapy and from OAD dual therapy to OAD dual therapy+basal insulin states. Kahn et al., (2006) found that 21% of patients failed to achieve their therapeutic goals after 5 years in metformin monotherapy [16]. In addition, Rascati et al., (2013) estimated that 23.6% of patients using metformin+SU dual therapy progressed to OAD dual therapy+basal insulin after 59 months in treatment [17].

**Table 1:** Health outcomes used in study model

Variables (Annual Rate)	Value^a^	References
Treatment failure		
Metformin monotherapy	0.046	Kahn et al., 2006 [16]
Metformin+dipeptidyl peptidase-4 inhibitor	0.013	Rascati et al., 2013 [17] and Bergenheim et al., 2012 [12]
Metformin+sulfonylurea	0.053	Rascati et al., 2013 [17]
Death rate		
60–70 years	0.021	Zhuo et al., 2014 [19]
71–80 years	0.051	Zhuo et al., 2014 [19]
Over 81 years	0.107	Zhuo et al., 2014 [19]
Death hazard ratio of Metformin+SU to Metformin+DPP-4i	1.850	Morgan et al., 2014 [20]
Hypoglycemia		
Severe hypoglycemia among patients with Metformin+SU	0.016	Goke et al., 2010 [9]
Hypoglycemia with medical assistance among patients with Metformin+SU	0.009	Goke et al., 2010 [9]
Severe hypoglycemia among patients with insulin glargine	0.010	The Origin Trial Investigators, 2012 [21]
Weight gain in the first year after starting SU	0.510	Bergenheim et al., 2012 [12]
Myocardial infarction		
Metformin monotherapy	0.004	Kahn et al., 2006 [16]
Metformin+dipeptidyl peptidase-4 inhibitor	0.004	Gitt et al., 2013 [11]
Metformin+sulfonylurea	0.000	Gitt et al., 2013 [11]
Insulin glargine	0.009	The Origin Trial Investigators, 2012 [21]
Heart failure		
Metformin monotherapy	0.003	Kahn et al., 2006 [16]
Metformin+dipeptidyl peptidase-4 inhibitor	0.017	Gitt et al., 2013 [11]
Metformin+sulfonylurea	0.020	Gitt et al., 2013 [11]
Insulin glargine	0.009	The Origin Trial Investigators, 2012 [21]
Stroke		
Metformin monotherapy	0.003	Kahn et al., 2006 [16]
Metformin+dipeptidyl peptidase-4 inhibitor	0.002	Gitt et al., 2013 [11]
Metformin+sulfonylurea	0.020	Gitt et al., 2013 [11]
Insulin glargine	0.009	The Origin Trial Investigators, 2012 [21]

^a^ Probability during certain period was converted to the rate per 1 year using following equation. (The rate was assumed to be constant over that period) $Rate=1-{\left(\frac{initial\kern0.17em probability- probability\kern0.17em change}{initial\kern0.17em probability}\right)}^{\frac{1}{years}}$

Parchman and Wang, (2012) found that the rate of insulin initiation had a statistically significant and positive association with the A1c increasing rate [18]. More specifically, Bergenheim et al., (2012) [12] found that patients using metformin+DPP-4i had four times lower A1c increasing rates than those using metformin+SU. Thus, we assumed that the annual treatment failure rate of metformin+DPP-4i was four times lower than metformin+SU.

Death rates for 60 to 70 years old, 71 to 80 years, and 81 to 85 years old groups were derived from the literature [19]. The hazard ratios of death in patients using metformin+SU and metformin+DPP-4i were also drawn from the literature [20]. Hypoglycemia probabilities in patients using metformin+SU and metformin+DPP-4i were extracted from the results of a 52-week randomized clinical trial [9]. We considered only severe hypoglycemia and hypoglycemia events requiring medical assistance to estimate direct health care costs. The probability of severe hypoglycemia for a patient using OAD + basal insulin was collected from the ORIGIN trial [21]. Weight gain data in patients using SU were derived from a previous study [12]. We assumed that use of metformin, DPP-4i and basal insulin were not associated with a significant weight gain [10].

Likewise, annual cardiovascular complication rates (i.e., myocardial infarction, heart failure and stroke) in patients using metformin monotherapy, OAD dual therapy, and OAD-basal insulin therapy for each health state described in the Markov model were derived from previous published clinical trial studies [11, 16, 21]. We assumed that the probability of treatment failure, hypoglycemia and cardiovascular complications remained constant through the study period with the exception of death rates which gradually increase with age. The proportion of patients in each state and cycle was calculated using the transition matrix (Tables 2 and 3).

**Table 2:** Transition matrix for the treatment pathway metformin+dipeptidyl peptidase-4 inhibitor

		To t + 1			
		Metformin monotherapy	Metformin +DPP-4i	Metformin +DPP-4i + Basal insulin	Death
From t	Metformin monotherapy	#	0.046	0	60–70 years; 0.021 71–80 years; 0.051 81–85 years; 0.107
	Metformin +DPP-4i	0	#	0.013	60–70 years; 0.021 71–80 years; 0.051 81–85 years; 0.107
	Metformin +DPP-4i + Basal insulin	0	0	#	60–70 years; 0.021 71–80 years; 0.051 81–85 years; 0.107
	Death	0	0	0	1

Acronyms: DPP-4i-dipeptidyl peptidase-4 inhibitors

**Table 3:** Transition matrix for the treatment pathway metformin+sulfonylureas

		To t+1			
		Metformin monotherapy	Metformin +SU	Metformin +SU + Basal insulin	Death
From t	Metformin monotherapy	#	0.046	0	60–70 years; 0.021 71–80 years; 0.051 81–85 years; 0.107
	Metformin +SU	0	#	0.053	60–70 years; 0.021 × 1.85 (HR) 71–80 years; 0.051 × 1.85 (HR) 81–85 years; 0.107 × 1.85 (HR)
	Metformin +SU + Basal insulin	0	0	#	60–70 years; 0.021 71–80 years; 0.051 81–85 years; 0.107
	Death	0	0	0	1

Acronyms: SU-sulfonylureas, HR-hazard ratio

The main study outcome was the number of life-years gained over the study time horizon. In order to estimate life-years gained, all life-years for patients in every state, with the exception of death, were aggregated by year and discounted. The incremental life-years gained were estimated as the difference in life-years gained between the two interest therapeutic alternatives metformin+DPP-4i and metformin+SU.

Direct health-care costs related to T2D, which included drug costs and treatment costs for diabetes related medical events such as hypoglycemia, weight gain and cardiovascular events were obtained from the US health care payer perspective. Direct health care cost input data were derived from the literature (Table 4). Indirect and intangible costs related to the disease were not included in the study model.

**Table 4:** Direct health care annual costs (2015 USD)

	Average annual costs	References
Health care costs (per episode/year)		
Myocardial infarction	$18627	Bergenheim et al., 2012 [12]
Heart failure	$14118	Bergenheim et al., 2012 [12]
Stroke	$7939	Bergenheim et al., 2012 [12]
Hypoglycemia events requiring medical assistance	$199	Bergenheim et al., 2012 [12]
Severe hypoglycemia event	$146	Bergenheim et al., 2012 [12]
Weight gain	$289	Bergenheim et al., 2012 [12]
Drug cost (per patient/year)		
Metformin, generic drug	$24	NADAC (January 2015) [22]
Dipeptidyl peptidase-4 inhibitor, brand	$3500 ($3401; $3599)	NADAC (January 2015) [22]
Sulfonylurea (glipizide), generic	$16	NADAC (January 2015) [22]
Insulin glargine, brand	$3646	NADAC (January 2015) [22]

All drug costs and direct health-state costs were expressed in 2015 US dollars ($) per patient/year

Antidiabetic drug costs data were collected from the National Average Drug Acquisition Cost (NADAC) dataset [22]. We used generic NADAC for metformin and SU (glipizide). The cost of DPP-4i was estimated as the average NADAC of sitagliptin, saxagliptin, linagliptin, and alogliptin. We also used the NADAC for basal insulin glargine pen type. Needle cost for insulin glargine pen was estimated at 80% of the average wholesale price (AWP). AWP data were derived from the online version of the RedBook [23]. Annual prescription drug cost was calculated based on the defined daily dose (DDD) from WHO Collaborating Centre for Drug Statistics Methodology [24]. The direct health care cost in each state, with the exception of death, was estimated for each year through the study time horizon. Base-case health care costs in each state was calculated multiplying the probability of each episode and unit cost (Table 5). All costs were adjusted to 2015 US dollars using the all urban consumers, not seasonally adjusted, US city average, all items, consumer price index (CPI) [25]. Both costs and outcomes were discounted at a 3% annual discount rate.

**Table 5:** Base-case direct health care cost results of five treatment strategies

	Medical Costs	Costs per Hypoglycemia Event	Costs per Cardiovascular Events	Weight Gain Costs (transition costs^a^)	Total Costs (without transition costs^a^)
Metformin monotherapy	$24	$0	$141	$0	$165
Metformin+DPP-4i	$3524	$0	$330	$0	$3854
Metformin+DPP-4i + Basal insulin	$7170	$1	$366	$0	$7537
Metformin+SU	$40	$4	$441	$148	$486
Metformin+SU + Basal insulin	$3686	$1	$366	$0	$4054

Acronyms: DPP-4i-dipeptidyl peptidase-4 inhibitors, SU-sulfonylureas

^a^ Transition cost was added only one time when patients transitioned from the metformin monotherapy state to the metformin+SU state

### Cost-effectiveness analysis

The cost-effectiveness analysis (CEA) of metformin+DPP-4i vs. metformin+SU in patients with T2D was conducted from the US health care payer perspective. The cost and life-years gained over the 25-year time horizon were estimated for each treatment pathway. A cost-effectiveness ratio (CER) was employed to calculate the cost per life-year gained for each treatment strategy. The lowest cost per life-year treatment was considered as the reference therapy. When a treatment had a greater cost and effectiveness in relation to the reference an incremental cost-effectiveness ratio was performed to determine the additional cost to obtain one life-year. Incremental cost-effectiveness ratio (ICER) was estimated for metformin+DPP-4i compared to metformin+SU.

### One-way and probabilistic sensitivity analyses

The impact of parameter uncertainty was explored by one-way sensitivity analysis on each model parameter. Results of the one-way sensitivity analysis were expressed as tornado charts. Values for treatment failure rates, hypoglycemia events probabilities, weight gain rates, cardiovascular events rates, and costs were changed by ±25% from the base-case. The cost of insulin glargine was changed by ±20%. Death rates and the death hazard ratios were changed by ±10%. The one-way sensitivity analysis was also conducted to compare differences in study results using 20 and 30 year time horizons. The sensitivity analyses also included a scenario in which there was no difference in cardiovascular event rates after 2 years from initiation of metformin+DPP-4i and metformin+SU [26, 27].

Inzucchi et al., (2015) found that the mean (standard deviation) age at the start of the antidiabetic treatment was 57.4 (11.7) years [28]. The average time to insulin initiation was 1.94–20.7 years depending on basal treatment. Machado-Alba et al., (2015) found that mean age at the start of oral antidiabetic therapy in patients with type 2 diabetes mellitus was 63.4 years [29]. After 5 years, 26.1% initiated insulin therapy. Roussel et al., (2016) found that the average age (standard deviation) of insulin therapy initiation in patients with type 2 diabetes mellitus (T2DM) was 67.5 (14.2) years [30]. In one-way sensitivity analyses, we changed the patient age at start of the antidiabetic treatment metformin monotherapy from 60 years in the base case to 55 and 65 years.

A probabilistic sensitivity analysis was conducted to investigate the combined impact of uncertainty of the variables included in the analysis. Random values were drawn from the chosen distributions as a second-order Monte-Carlo simulation of 1000 patients to estimate the mean and 95% confidential intervals (CI) of costs and life-years gained. All parameters in the model had correspondingly appropriate distributions. Costs were randomly drawn from a gamma distribution; hazard ratio were randomly sampled from a lognormal distribution. Likewise, binominal data, such as hypoglycemia probabilities and cardiovascular event rates were randomly drawn from a beta distribution. Multinomial data, such as transition probabilities in the metformin monotherapy and the OAD dual therapy states, were randomly sampled from a Dirichlet distribution [31].

The ICER was recalculated based on the patient age at start of the antidiabetic treatment, the average incremental costs and life-years gained derived from the probabilistic sensitivity analysis. The simulation output was presented using a cost-effectiveness plane. A cost-effectiveness acceptability curve (CEAC) was also plotted to summarize the uncertainty in the cost-effectiveness estimates.

## Results

### Base-case analysis

Diabetic-related annual average costs and life-years gained after discounting were $18,853 and 12.42 years, respectively for patients in the metformin+DPP-4i treatment pathway. Patients in the metformin+SU treatment pathway incurred in a lower annual average costs per patient ($7004) and gained on average a lower number of life-years (11.81 years) (Table 6). The incremental costs and life-years gained for metformin+DPP-4i compared to metformin+SU treatment pathways were $11,849 and 0.61 years, respectively. Thus, the ICER was $19,420 per life-year gained for patients in the metformin+DPP-4i treatment pathway.

**Table 6:** Base-case cost and effectiveness results of treatment strategies (per patient)

Discounted (3% annual discount rate)					
Second-line agent add-on to Metformin	Total		Incremental		
	Costs	LYs gained	Costs	LYs gained	ICER
Sulfonylurea	$7004	11.81			
Dipeptidyl peptidase-4 inhibitor	$18853	12.42	$11849	0.61	$19420
Undiscounted					
Sulfonylurea	$10501	15.68			
Dipeptidyl peptidase-4 inhibitor	$28013	16.70	$17512	1.02	$17170

All costs were expressed in 2015 US dollars ($)

Acronyms: LY-Life-year, CER-cost-effectiveness ratio (equal to cost/LY), ICER- incremental cost-effectiveness ratio (equal to incremental cost/incremental LYs)

### One-way and probabilistic sensitivity analyses

**Fig. 2:**
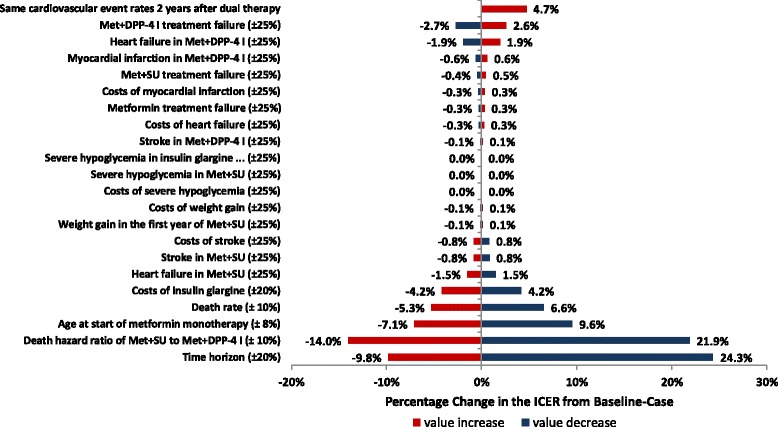
Tornado diagram of one-way sensitivity analysis (percentage changes in the ICER from base-case), Acronyms; Met-metformin, DPP-4i-dipeptidyl peptidase-4 inhibitor, SU-sulfonylurea

One-way sensitivity analysis was conducted by varying the range of values in the base-case to determine potential impacts on the results. The percentage changes in ICER from base-case are presented in the tornado graph (Fig. 2). Time horizon, death hazard ratio and age at start of metformin monotherapy, and death rate parameters in the model had largest impact on the model results. The results of the analysis did not change significantly when varying estimates used in the base-case scenario (Table 7). Results for the base-case scenario were not sensitive to changes in the costs of insulin glargine, treatment failure rates, costs and rates of cardiovascular events, or the costs and probabilities of severe hypoglycemia, and weight gain. Results for the base-case scenario were not sensitive either to changes in the cardiovascular event rates of metformin+DPP-4i and metformin+SU.

**Table 7:** Results of one-way sensitivity analyses for base-case scenario

	Values	Estimated ICER
Base Case		$19420
Death hazard ratio of Met+SU to Met+DPP-4i	1.67 (−10%)	$23760
Death hazard ratio of Met+SU to Met+DPP-4i	2.04 (+ 10%)	$16760
Metformin treatment failure	0.035 (−25%)	$19440
Metformin treatment failure	0.058 (+ 25%)	$19560
Met+DPP-4i treatment failure	0.010 (−25%)	$18970
Met+DPP-4i treatment failure	0.017 (+ 25%)	$20010
Met+SU treatment failure	0.040 (−25%)	$19410
Met+SU treatment failure	0.067 (+ 25%)	$19590
Severe hypoglycemia in Met+SU	0.012 (−25%)	$19500
Severe hypoglycemia in Met+SU	0.020 (+ 25%)	$19500
Severe hypoglycemia in insulin glargine triple therapy	0.008 (−25%)	$19500
Severe hypoglycemia in insulin glargine triple therapy	0.013 (+ 25%)	$19500
Weight gain in the first year of Met+SU	0.383 (−25%)	$19520
Weight gain in the first year of Met+SU	0.638(+ 25%)	$19470
Myocardial infarction in Met+DPP-4i	0.003(−25%)	$19380
Myocardial infarction in Met+DPP-4i	0.005(+ 25%)	$19620
Heart failure in Met+DPP-4i	0.013(−25%)	$19120
Heart failure in Met+DPP-4i	0.021(+ 25%)	$19880
Stroke in Met+DPP-4i	0.002(−25%)	$19470
Stroke in Met+DPP-4i	0.003(+ 25%)	$19520
Heart failure in Met+SU	0.015(−25%)	$19790
Heart failure in Met+SU	0.025(+ 25%)	$19210
Stroke in Met+SU	0.015(−25%)	$19660
Stroke in Met+SU	0.025(+ 25%)	$19340
Costs of myocardial infarction	$13970(−25%)	$19430
Costs of myocardial infarction	$23284(+ 25%)	$19570
Costs of heart failure	$10589(−25%)	$19450
Costs of heart failure	$17648(+ 25%)	$19550
Costs of stroke	$5954(−25%)	$19660
Costs of stroke	$9924(+ 25%)	$19340
Costs of severe hypoglycemia	$110(−25%)	$19500
Costs of severe hypoglycemia	$183(+ 25%)	$19500
Costs of weight gain	$217(−25%)	$19520
Costs of weight gain	$361(+ 25%)	$19470
Costs of insulin glargine	$2917(−20%)	$20320
Costs of insulin glargine	$4375(+ 20%)	$18680
Death rate	0.019 (age 60–70) / 0.046 (age 71–80) / 0.096 (age 81–85) (−10%)	$20780
Death rate	0.024 (age 60–70) / 0.057 (age 71–80) / 0.118 (age 81–85) (+ 10%)	$18470
Time horizon	20 years (− 20%)	$24250
Time horizon	30 years (+ 20%)	$17580
Same cardiovascular event rates from 2 years after dual therapy	MI; 0.004 / HF; 0.02 / Stroke; 0.02	$20420
Age at start of metformin monotherapy	55 (−8%)	$21360
Age at start of metformin monotherapy	65 (+ 8%)	$18120

Acronyms: SU-sulfonylurea, DPP-4i-dipeptidyl peptidase-4 inhibitor, Met; metformin, MI-myocardial infarction, HF-heart failure

The ICER increased to $24,250 per life-year gained (+ 24.3%) when the time horizon decreased to 20 years. Conversely, the ICER decreased to $17,580 per life-year gained (− 9.8%) when the time horizon increased to 30 years. In addition, a 10% decrease in the death hazard ratio resulted in a 21.9% increase in the ICER ($23,760). A 10% increase in the death hazard ratio resulted in a 14.0% decrease in the ICER ($16,760). Assuming that patients start metformin monotherapy at age of 55 increased ICER to $21,360 (9.6%); whereas, starting antidiabetic treatment at age of 65 decreased ICER to $18,120 (− 7.1%).

The average results of the probabilistic sensitivity analysis yielded $11,786 incremental costs and 0.59 incremental life-year gained for patients using metformin+DPP-4i compared to alternative metformin+SU treatment pathway (Table 8). The ICER in the probabilistic sensitivity analysis was $19,980 per life-year gained. The difference between the probabilistic sensitivity analysis and the base-case strategy was $63 in incremental costs and 0.02 additional life-years gained.

**Table 8:** Probabilistic Sensitivity Analysis

Second-line agent add-on to Metformin	Average Total		Average Incremental		
	Costs	Life Years Gained	Costs	Life Years Gained	ICER
Sulfonylurea	$7004 (±316.52)	11.93 (±0.07)			
Dipeptidyl peptidase-4 inhibitor	$18790 (±1008.70)	12.52 (±0.07)	$ 11786 (±976.92)	0.59 (±0.02)	$19980

**Fig. 3:**
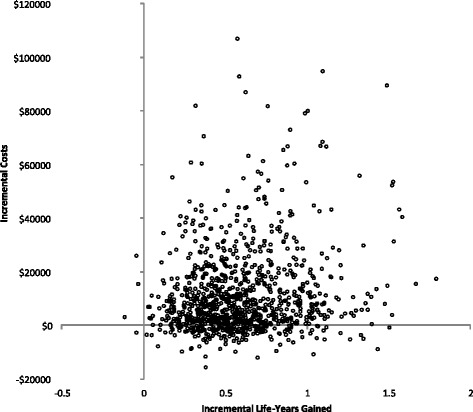
Cost-Effectiveness Plane, Scatter plots showing the 1000 cases of differences in costs and in the life-year gained from the trial data using 1000 bootstrap replicates

The uncertainty surrounding the expected costs and outcomes associated with metformin+DPP-4i compared with metformin+SU is illustrated in Fig. 3. The incremental cost-effectiveness plane shows the trade-offs in the northeast (i.e., positive costs and positive effects) and southeast quadrants (i.e., negative costs and positive effects).

**Fig. 4:**
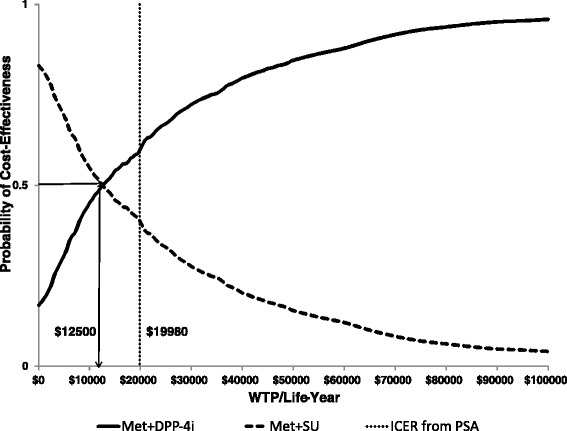
Cost-effectiveness Acceptability Curve

The CEAC indicates that metformin+DPP-4i and metformin+SU would have the same probability of being the most cost-effective treatment for a WTP threshold of $12,500 per life-year gained; after exceeding this threshold the probability of metformin+DPP-4i being the most cost-effective treatment pathway approaches to 1 (Fig. 4).

## Discussion

This study assessed the long-term cost-effectiveness of metformin+DPP-4i compared with metformin+ SU treatment pathways as second-line therapy from the US health care payer perspective. In the base-case results, the total costs and life-years gained were higher for metformin+DPP-4i than for the metformin+SU treatment pathway.

The results from the probabilistic sensitivity analyses were similar to those of the base-case results. The results of the one-way sensitivity analysis showed that the main factors impacting on the ICER were time horizon and death hazard ratio from metformin+SU to metformin+DPP-4i. The probability that the DPP-4i treatment pathway would become the cost-effective alternative compared to metformin+SU increases as the WTP per life-year threshold increases. When the WTP per life-year equals $12,500 per life-year, the probability of the DPP-4i treatment pathway to be the most cost-effective alternative become 0.5.

The results of this study differ from two previous cost-effectiveness studies conducted in the US that compared metformin+DPP-4i and metformin+SU as a second line therapy. Bergenheim et al., (2012) compared metformin+saxagliptin with metformin+glipizide for the treatment of T2D [12]. The authors concluded that metformin+DPP-4i was a cost-effective second-line therapy in the US. Some methodological differences between Bergenheim et al., (2012) and this study are worth mentioning. Unlike this study, Bergenheim et al., (2012) did not include metformin monotherapy as the base-case therapy in T2D treatment. They did not consider either the treatment alternative OAD + insulin after metformin+SU and metformin+DPP-4i treatment failure. Bergenheim et al., (2012) considered the use of insulin as the rescue therapy when the A1c level was higher than 7.5%. They set up the patient lifetime as the study time horizon and used a Cardiff Long-term Cost Utility Model for the cost-effectiveness estimation. Bergenheim et al., (2012) estimated metformin+saxagliptin treatment pathway had a $2772 higher costs (2009 USD) and 2.65 greater QALYs (ICER was $1047 per QALY) compared to metformin+glipizide alternative. A life-time horizon allows to better understand the burden of the disease on patients with T2D inclusive of all treatment alternatives and related costs.

In addition, the difference in costs between Bergenheim et al., (2012) and this study is driven by the difference in the generic DPP-4i prices estimation. In this study we used NADAC prices of branded DPP-4i. Conversely, Bergenheim et al., (2012) assumed that generic DPP-4i would enter the market in a 10 year-time frame and that generic DPP-4i prices would be 16% of the corresponding brand name drug price. Nevertheless, prices of generic drugs are set up as 94% of brand name drug prices when there is only one generic competitor in the market [32]. A decrease in the price of generic drugs to 16% of the price of brand name drugs is observed only when there are several generic competitors in the market. Therefore, Bergenheim et al., (2012) may underestimate DPP-4i generic drug prices.

Zhang et al., (2014) compared metformin+SU, metformin+DPP-4i, metformin+GLP-1 receptor agonists, and insulin as the second-line therapy using a Markov model with 10 A1c states [8]. Zhang et al., (2014) only assessed the medication cost; Authors did not include the costs of medical events related with diabetic complications. Zhang et al., (2014) did not include either in the analysis the costs associated with severe hypoglycemia. Furthermore, they assumed that the termination state was either the first diabetes-related complication or death.

Zhang et al., (2014) set up three different A1c goals (i.e., 6.5, 7, and 8%) to evaluate the impact of glycemic control goals on patients with diabetes. Like in our study, they considered insulin triple therapy after dual therapy failure, but they assumed that patients initiated insulin therapy only after exceeding the A1c goals. This assumption lead to the initiation of insulin therapy only after 1.59 to 2.76 years after onset T2D diagnosis. Nevertheless, assuming that insulin initiation is based on patients’ A1c levels may lead to overestimate insulin initiating rate due to well document barriers for the patient’s and provider’s to start insulin therapy [33–35]. In addition, the timeline for second line therapy before insulin therapy may not be long enough to show clinically meaningful differences in outcomes associated with the use of alternative second line therapies. Last, Zhang et al., (2014) assumed that the first diabetes-related complication and death were termination states, resulting in lower rates of diabetic complications and costs than this study estimations. Zhang et al., (2014) concluded that the life-years and QALYs until the first event were similar in the four treatment pathways and that metformin+SU had similar outcomes and lower drug costs compared to the assessed treatment alternatives.

### Limitations

The Markov model used in this study does not intent to represent the actual clinical progression of patients with diabetes but to assess differences in two alternative therapy pathways under a defined set of assumptions. Thus, study results should be interpreted taking into consideration some limitations. Adult patients may develop T2D at any time during their life. This study assumed patients entered the model at age 60 years old. Study results are not generalizable to other T2D therapy initiation ages.

The Markov model employed in this study, assessed alternative pharmacological treatment pathways for T2D. To define the Markov states, this study model included most common drug therapies for the treatment of T2D instead of conventional health states, such as patient’ A1c level or disease progress status [36, 37]. Therefore, in this study transition probabilities did not depend on changes in A1c or disease progressions but on treatment failure rates observed in prior studies in patients with T2D.

We set the study time horizon at 25 years for the base-case because the survival data were available only until patients reach 85-years old. The death hazard ratio was estimated based on data drawn from a two-year trial results. Therefore, a more robust model would include death rates data for patients with diabetes for a longer time horizon [38].

Due to scarcity of studies some clinical input data were derived from trials outside of the US. Treatment failure rates for metformin were derived from studies conducted in the US, Canada, and the European Union (EU). Death hazard ratio for metformin+SU was derived from a study conducted in the United Kingdom and cardiovascular complication rates for dual therapy were derived from the studies conducted in the EU. Hypoglycemia data were derived from an international randomized clinical trial. Hypoglycemic events might lead to changes in medication. Future studies may include more treatment alternatives to account for changes in medication. Representativeness of study results may improve in the future using ongoing long-term comparative effectiveness studies conducted in the US in patients with T2D as model inputs [39]. Additionally, the study did not account for changes in clinical practice that have occurred after the publication of some of the studies used to derive the clinical input data.

Probabilities of treatment failure, hypoglycemia and cardiovascular complications were derived from studies with a limited time horizon. In addition, we assumed that the rates of cardiovascular events and treatment failure, and insulin dose remained constant through the study time horizon. Hypoglycemia rate data were derived from a trial which included patients with prediabetes. Thus, the hypoglycemia rate may be overestimated.

Cost-effectiveness estimations included only hypoglycemia, weight gain and cardiovascular events- related costs. While these outcomes have been documented as the main outcomes differences between DPP-4i and SU other differences in outcomes between these treatment alternatives may exist [9, 40]. Weight gain caused by insulin glargine was not considered in this study. Including different weight gain rates for each treatment pathway would yield more robust estimations but it would significantly increase the complexity of the Markov model. We conducted a sensitivity analysis for several key study measures including weight gain rates and study results did not change significantly. Microvascular complications, such as amputation, blindness or end state renal disease were not included either in the study because these outcomes are associated with uncontrolled blood glucose level and not with the use of specific drugs. Acute treatment costs for cardiovascular events were included in the CEA. Thus, medical costs for T2D related cardiovascular events could be underestimated.

We assumed that patients were adherent to antidiabetic medications when estimating the outcomes and drug costs. High medication costs may impact on the DPP-4i treatment adherence. Likewise, the risk of hypoglycemia may impact on the adherence of SU and insulin. Fixed-dose combination drugs were not considered when estimating medication costs. Self-monitoring of blood glucose related costs were not included in the CEA.

This study assessed metformin+SU and metformin+DPP-4i treatment pathways; other treatment alternatives are marketed in the US such as newly FDA approved sodium glucose cotransporter-2 inhibitors and GLP-1 receptor agonists. Last, study model did not include triple oral or dual oral plus non-insulin injectable treatment alternatives before initiating insulin and prandial insulin option. Future studies may include more complex Markov models to compare the cost-effectiveness of all currently available T2D treatment pathways. Future studies might consider the use of quality of life adjusted years as the study outcomes.

In spite of these limitations, this study has important strengths. Our study assessed three alternative treatment pathways during a long-term time horizon to better capture progression of the disease overtime. In addition, this study comprehensively assessed all prescription drug and health care costs related with diabetes complications. Furthermore, this study used insulin initiating rate to reflect both patients and providers decision making process to start insulin therapy.

## Conclusions

This study assessed the cost-effectiveness of most commonly recommended in clinical guidelines T2D alternative long-term treatment pathways. The treatment pathway with DPP-4i as the second-line therapy was cost-effective compared to SU from the US health care payer perspective. The results of the one way and probabilistic sensitive analyses indicate that study findings are not sensitive to changes in the parameters used in the model. More studies assessing the cost-effectiveness of all long-term alternative T2D treatment pathways marketed in the US are needed.

## Abbreviations

A1c: Glycated hemoglobin
AWP: Average wholesale price
CEA: Cost-effectiveness analysis
CEAC: Cost-effectiveness acceptability curve
CPI: Consumer price index
DDD: Defined daily dose
EU: European union
FDA: U.S. Food and drug administration
ICER: Incremental cost-effectiveness ratio
NADAC: National average drug acquisition cost
OAD: Oral antidiabetic drugs
T2D: Type 2 diabetes
US: United States
WHO: World Health Organization
WTP: Willingness to pay
